# Improvement in left ventricular ejection fraction after pharmacological up-titration in new-onset heart failure with reduced ejection fraction

**DOI:** 10.1007/s12471-021-01591-6

**Published:** 2021-06-14

**Authors:** J. F. Nauta, B. T. Santema, M. H. L. van der Wal, A. Koops, J. Warink-Riemersma, K. van Dijk, F. Inkelaar, S. Prückl, J. Suwijn, V. M. van Deursen, W. C. Meijers, J. Coster, B. D. Westenbrink, R. A. de Boer, Y. Hummel, J. van Melle, D. J. van Veldhuisen, P. van der Meer, A. A. Voors

**Affiliations:** Department of Cardiology, University of Groningen, University Medical Centre Groningen, Groningen, The Netherlands

**Keywords:** Heart failure, Guideline adherence, Target doses, Multidisciplinary care, Heart failure with reduced ejection fraction

## Abstract

**Objective:**

Recent studies have reported suboptimal up-titration of heart failure (HF) therapies in patients with heart failure and a reduced ejection fraction (HFrEF). Here, we report on the achieved doses after nurse-led up-titration, reasons for not achieving the target dose, subsequent changes in left ventricular ejection fraction (LVEF), and mortality.

**Methods:**

From 2012 to 2018, 378 HFrEF patients with a recent (< 3 months) diagnosis of HF were referred to a specialised HF-nurse led clinic for protocolised up-titration of guideline-directed medical therapy (GDMT). The achieved doses of GDMT at 9 months were recorded, as well as reasons for not achieving the optimal dose in all patients. Echocardiography was performed at baseline and after up-titration in 278 patients.

**Results:**

Of 345 HFrEF patients with a follow-up visit after 9 months, 69% reached ≥ 50% of the recommended dose of renin-angiotensin-system (RAS) inhibitors, 73% reached ≥ 50% of the recommended dose of beta-blockers and 77% reached ≥ 50% of the recommended dose of mineralocorticoid receptor antagonists. The main reasons for not reaching the target dose were hypotension (RAS inhibitors and beta-blockers), bradycardia (beta-blockers) and renal dysfunction (RAS inhibitors). During a median follow-up of 9 months, mean LVEF increased from 27.6% at baseline to 38.8% at follow-up. Each 5% increase in LVEF was associated with an adjusted hazard ratio of 0.84 (0.75–0.94, *p* = 0.002) for mortality and 0.85 (0.78–0.94, *p* = 0.001) for the combined endpoint of mortality and/or HF hospitalisation after a mean follow-up of 3.3 years.

**Conclusions:**

This study shows that protocolised up-titration in a nurse-led HF clinic leads to high doses of GDMT and improvement of LVEF in patients with new-onset HFrEF.

**Supplementary Information:**

The online version of this article (10.1007/s12471-021-01591-6) contains supplementary material, which is available to authorized users.

## What’s new?

- Guideline-directed medical therapies (GDMT) are not always initiated or up-titrated to target doses, and reasons for not doing so are often not reported.
- This study reports on the changes in left ventricular ejection fraction (LVEF) in the setting of a protocolised up-titration in a specialised nurse-led heart failure (HF) clinic.
- The reasons for not reaching recommended doses were recorded in all patients.
- This study shows that high doses of GDMT and improvement of LVEF can be achieved in specialised nurse-led HF clinics using a guideline-based up-titration protocol. This study therefore argues in favour of using this setting for patients with new-onset HF.

## Introduction

Heart failure (HF) is considered a chronic and often progressive disease. In the years following the diagnosis of HF a substantial proportion of patients develop left ventricular remodelling, which over time becomes maladaptive, and is characterised by increased dimensions of the left ventricle, thinner walls and decreased left ventricular ejection fraction (LVEF) [1]. However, reverse remodelling (marked by a reduction of LV dimensions and improvement in LVEF) can be achieved in selected patients [2]. In a recent study of 1160 patients with heart failure with reduced ejection fraction (HFrEF), LVEF showed a marked rise during the first year, followed by a relatively long plateau phase of up to a decade and then a subsequent slow decline [3]. One of the factors that is positively associated with reverse remodelling is up-titration of guideline-directed medical therapy (GDMT) [4].

In clinical trials and registries, a large majority of patients with HFrEF (80–90%) receive beta-blockers and/or angiotensin-converting-enzyme inhibitors/angiotensin receptor blockers (ACEIs/ARBs) (at any dose). However, up-titration of GDMT is notoriously difficult and many patients never receive adequate doses. In the CHAMP-HF registry of 2588 outpatients with HFrEF from the United States, the percentage of those receiving target doses of mineralocorticoid receptor antagonists (MRAs), beta-blockers, ACEIs/ARBs or angiotensin receptor-neprilysin inhibitor (ARNIs) after 12 months of follow-up was 27%, 22%, 10% and 3% respectively [5]. In the cross-sectional CHECK-HF registry of 34 HF outpatient clinics in the Netherlands, median achieved drug doses were 50% of the target dose for renin-angiotensin-system (RAS) inhibitors, 25% of the target dose for beta-blockers, and 25% of the target dose for MRAs [6].

Reasons for poor up-titration might be related to the healthcare system, patient preference, or medication side effects [7]. Common side effects such as fatigue, hypotension, renal dysfunction and hyperkalaemia can overlap with the syndrome of HF itself, further complicating treatment decisions.

The 2016 European Society of Cardiology (ESC) HF guidelines recommend multidisciplinary care management programmes for HF patients to improve survival and reduce the number of HF hospitalisations. One way to provide structured care to HF patients is the implementation of HF outpatient clinics led by specialised HF nurses. In a nurse-led disease management programme, nurses provide structured education on self-care and psychosocial care for patients and their family. Nurse-led care also improves the adoption of GDMT, and led to more favourable improvement in patient-reported outcomes and LVEF in a randomised controlled study [8]. Moreover, referral to a nurse-led HF clinic was associated with a lower risk of death in a Swedish national registry [9].

In the present study in a population of patients with newly diagnosed HFrEF, the doses achieved after up-titration in a nurse-led HF clinic were analysed, including the reasons for not achieving the guideline-recommended target dose. Furthermore, we assessed changes in LVEF after 9 months of follow-up, and studied HF hospitalisations and all-cause mortality.

## Methods

### Study population

We performed a retrospective longitudinal cohort study of consecutive patients with new-onset HF who presented to our tertiary care hospital between 2012 and 2018. Patients were included when they were referred to a specialised HF outpatient clinic, either after a first appointment with a cardiologist or 10 days after discharge following a first hospitalisation for HF. Patients had to be diagnosed with HF by a cardiologist no longer than 3 months before inclusion. In the outpatient clinic, GDMT was initiated and/or up-titrated by specialised HF nurses. Up-titration was done using pre-specified protocols based on the ESC guidelines for the diagnosis and treatment of HF (Electronic Supplementary Material, Table S1) [10, 11]. Furthermore, patients received education on self-care and had easy access to the HF clinic in the case of worsening HF. Clinical characteristics and doses of HF medication were recorded at the baseline and follow-up visits. We selected the visit that was performed 9 months after the baseline visit, or the visit that was closest to 9 months after baseline, to establish the success of up-titration and to assess changes in LVEF. We chose 9 months because patients were expected to be fully up-titrated by this time [12]. If patients had not achieved 100% of the guideline recommended target dose for either beta-blockers, ACEIs/ARBs/ARNIs or MRAs at this visit, the reason for incomplete up-titration was recorded. At the censor date of 1 September 2018, survival status and number of HF hospitalisations were recorded for every patient. This study complied with the Declaration of Helsinki and all national and local regulations as confirmed by the medical ethical evaluation committee. Patients were involved in the design and conduct of this research.

### Echocardiography

Echocardiography was performed as part of routine clinical care. A baseline echocardiogram was selected that was closest to the actual date of the baseline visit to the clinic. Patients were excluded if LVEF could not be reliably determined at baseline. A follow-up echocardiogram was selected closest to the 9‑months follow-up visit, and at least 6 months after the initial visit.

### Statistical analysis

Normally distributed data are presented as mean (standard deviation). Data that were not normally distributed are presented as median (25th percentile, 75th percentile). Intergroup differences were tested using one-way ANOVA for normally distributed data. The chi-square or Kruskal-Wallis test was used for not normally distributed data. We performed linear regression to analyse determinants of change in ejection fraction. We considered any baseline demographic, clinical, laboratory and medication characteristic that was deemed important based on clinical reasoning for our multivariable analysis. Subsequently, stepwise backward selection was performed to derive the final model. Crude and multivariable adjusted Cox proportional hazard models were performed to evaluate the effect of changes in LVEF on mortality. All analysis were performed in R version 3.6.0.

## Results

### Baseline characteristics

In our cohort of new-onset HFrEF patients, the mean age of the 378 patients was 65.5 (±14.1) years and 34.1% were women. Baseline characteristics are presented in Tab. 1. A total of 345 patients had a follow-up visit, and 278 patients had echocardiography performed both at baseline and follow-up.

**Table 1:** Baseline characteristics of patients with heart failure with reduced ejection fraction (*HFrEF*)

	HFrEF (*n* = 378)
Age	65.5 (14.1)
Women	129 (34.1%)
Systolic blood pressure	119 (20)
Body mass index	26.6 (5.3)
*NYHA class*	
– I	51 (14.3%)
– II	199 (55.7%)
– III	102 (28.6%)
– IV	5 (1.4%)
*ECG rhythm*	
– Sinus rhythm	248 (65.6%)
– Atrial fibrillation	106 (28.0%)
– Pacemaker	24 (6.4%)
ECG heart rate	80 (17)
ECG QRS	117.1 (30.8)
*Echo*	
– LVEF	27.8 (8.4)
– LVEDD	56.5 (8.1)
– TAPSE	18.2 (5.5)
*Medical history*	
– Ischaemic heart disease	143 (37.8%)
– Hypertension	114 (30.2%)
– Dilated cardiomyopathy	55 (14.6%)
– Diabetes mellitus	73 (19.3%)
– COPD	38 (10.1%)
– Atrial fibrillation	131 (34.7%)
– Peripheral artery disease	59 (15.6%)
– Cancer	74 (19.6%)
– Chronic inflammatory disease	38 (10.1%)
– Thyroid disease	21 (5.6%)
*Medication (baseline)*	
– Beta-blocker use	339 (89.9%)
– ACEI/ARB/ARNI use	330 (87.3%)
– MRA use	157 (41.5%)
– Diuretic use	265 (70.1%)
*Laboratory*	
– Sodium (mmol/l)	140 (138, 142)
– Potassium (mmol/l)	4.3 (4.0, 4.6)
– Creatinine (µmol/l)	93 (77, 117)
– eGFR (ml/min per 1.73 m^2^)	66 (51, 83)
– NT-proBNP (mmol/l)	1647 (697, 3746)

Data are presented as mean (standard deviation), number (percentage) or median (25th percentile, 75th percentile)

*NYHA* New York Heart Association, *ECG* electrocardiogram, *LVEF* left ventricular ejection fraction, *LVEDD* left ventricular end-diastolic diameter, *TAPSE* tricuspid annular plane systolic excursion, *COPD* chronic obstructive pulmonary disease, *ACEI* angiotensin-converting-enzyme inhibitor, *ARB* angiotensin receptor blocker, *ARNI* angiotensin-receptor-neprilysin inhibitor, *MRA* mineralocorticoid antagonist, *eGFR* estimated glomerular filtration rate, *NT-proBNP* N-terminal pro-B-type natriuretic peptide

Use of beta-blockers and RAS inhibitors at any dose was already high at the baseline visit, with 90% of patients with HFrEF using any dose of beta-blockers. For RAS inhibitors, 87% used any dose at baseline. Use of MRAs was lower, with 41% of patients with HFrEF using an MRA at baseline (Tab. 1).

### Up-titration and reasons for not reaching target dose

Of 345 HFrEF patients who were on medication, 69% reached ≥ 50% of the recommended dose of ACEIs/ARBs/ARNIs, 73% reached ≥ 50% of the recommended dose of beta-blockers, and 77% reached ≥ 50% of the recommended dose of MRAs (Tab. 2). Reasons for not achieving the target doses are presented in Tab. 3. For beta-blockers, the main reasons for not reaching the recommended target dose were hypotension (40.2%) and bradycardia (25.6%). For ACEIs/ARBs/ARNIs, the most important reason for not reaching the target dose was hypotension (54.0%), followed by renal dysfunction (14.7%) and hyperkalaemia (6.2%). The most important reason for not reaching the recommended dose of MRAs was that there was no longer an indication at the time of the follow-up visit because the patients’ functional status had improved to New York Heart Association (NYHA) functional class I. Patients who did not receive (the recommended dose of) a MRA at the time of the 9‑months visit, since there was no guidelines indication anymore, had a survival rate that was comparable to that of HFrEF patients who reached 100% of the target dose (3-year survival rate 88% vs 94% respectively). Patients with HFrEF who had a guideline indication for MRAs that received less than 100% of the target dose had considerably worse survival (79% at 3 years) than those on recommended doses.

**Table 2:** Baseline and follow-up doses of guideline-directed medical therapy in patients with heart failure with reduced ejection fraction (*HFrEF*)

	HFrEF	
	Baseline	Follow-up
*Beta-blocker*	*n* = 309	*n* = 322
– 1–49%	116 (38%)	87 (27%)
– 50–99%	140 (45%)	146 (45%)
– ≥ 100%	52 (17%)	90 (28%)
*ACEI/ARB/ARNI*	*n* = 300	*n* = 306
– 1–49%	128 (42%)	94 (31%)
– 50–99%	120 (40%)	80 (26%)
– ≥ 100%	53 (18%)	133 (43%)
*MRA*	*n* = 146	*n* = 200
– 1–49%	2 (1%)	6 (3%)
– 50–99%	74 (51%)	78 (39%)
– ≥ 100%	70 (48%)	116 (58%)

*ACEI* angiotensin-converting-enzyme inhibitor, *ARB* angiotensin receptor blocker, *ARNI* angiotensin-receptor-neprilysin inhibitor, *MRA* mineralocorticoid antagonist

**Table 3:** Reasons for not initiating or further up-titrating guideline-directed medical therapy in patients with heart failure with reduced ejection fraction (*HFrEF*)

	Beta-blocker (*n* = 255)	ACEI/ARB/ARNI (*n* = 212)	MRA (*n* = 229)
No longer indicated (NYHA I)	21 (8.3%)	11 (5.2%)	89 (38.9%)
Hypotension	104 (40.8%)	117 (55.5%)	41 (17.9%)
Bradycardia	68 (26.8%)	0 (0.0%)	0 (0.0%)
Renal dysfunction	0 (0.0%)	32 (15.2%)	30 (13.1%)
Still in up-titration phase	14 (5.5%)	11 (5.2%)	29 (12.7%)
Hyperkalaemia	0 (0.0%)	13 (6.2%)	18 (7.9%)
Patient preference	10 (3.9%)	6 (2.8%)	10 (4.4%)
Fatigue	9 (3.5%)	0 (0.0%)	0 (0.0%)
Cough	0 (0.0%)	7 (3.3%)	0 (0.0%)
Cold extremities	7 (2.8%)	2 (0.9%)	0 (0.0%)
Other negative chronotropic medication	5 (2.0%)	0 (0.0%)	0 (0.0%)
Gynaecomastia	0 (0.0%)	0 (0.0%)	3 (1.3%)
Atrioventricular block	3 (1.2%)	0 (0.0%)	0 (0.0%)
Headache	2 (0.8%)	1 (0.5%)	0 (0.0%)
Other	1 (0.4%)	2 (0.9%)	0 (0.0%)
Not noted	1 (0.4%)	2 (0.9%)	2 (0.9%)
Physician decision (normalisation of LV function)	1 (0.4%)	2 (0.9%)	0 (0.0%)
Angio-oedema	0 (0.0%)	1 (0.5%)	0 (0.0%)
Gastrointestinal complaints	0 (0.0%)	1 (0.5%)	1 (0.4%)
Non-compliance	0 (0.0%)	1 (0.5%)	0 (0.0%)
Erectile dysfunction	2 (0.8%)	0 (0.0%)	0 (0.0%)
Pregnancy	1 (0.4%)	1 (0.5%)	1 (0.4%)
Renal artery stenosis	0 (0.0%)	1 (0.5%)	0 (0.0%)
Airway reactivity	1 (0.4%)	0 (0.0%)	0 (0.0%)
Cognitive or behavioural effect	1 (0.4%)	0 (0.0%)	0 (0.0%)
Dobutamine continuous infusion	1 (0.4%)	0 (0.0%)	0 (0.0%)
Elevated liver enzymes	0 (0.0%)	0 (0.0%)	1 (0.4%)
Fluid retention	1 (0.4%)	0 (0.0%)	0 (0.0%)
Gout	0 (0.0%)	0 (0.0%)	1 (0.4%)
Hypoglycaemia	1 (0.4%)	0 (0.0%)	0 (0.0%)
Itching	0 (0.0%)	0 (0.0%)	1 (0.4%)
Tingling sensation	1 (0.4%)	0 (0.0%)	0 (0.0%)
Vaginal bleeding	0 (0.0%)	0 (0.0%)	1 (0.4%)

*ACEI*  angiotensin-converting-enzyme inhibitor, *ARB* angiotensin receptor blocker, *ARNI* angiotensin-receptor-neprilysin inhibitor, *MRA* mineralocorticoid antagonist, *NYHA* New York Heart Association class,* LV* left ventricular

### Improvement in ejection fraction

**Fig. 1:**
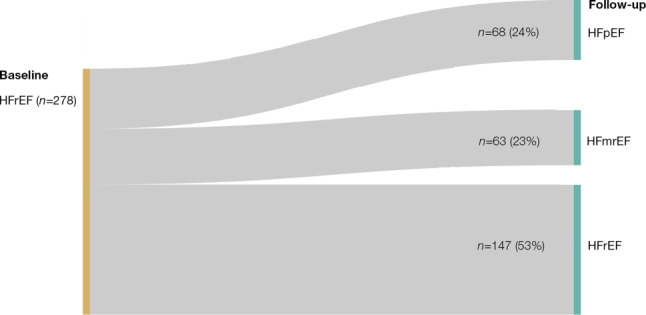
Flow diagram of changes in left ventricular ejection fraction in 278 patients with new-onset heart failure with reduced ejection fraction (*HFrEF*). *HFpEF* heart failure with preserved ejection fraction, *HFmrEF* heart failure with mid-range ejection fraction

During a median follow-up of 9 months, improvement from HFrEF to HF with mid-range EF/HF with preserved EF was seen in 131 of 344 of patients (35%). Fig. 1 shows a flow diagram of changes in LVEF categories. LVEF improved from a mean of 27.6% at baseline to 38.8% at follow up (+11.2%). Multivariable predictors of improvement of LVEF are presented in Tab. 4. Baseline LVEF was the strongest predictor of subsequent improvement. In addition, older age, non-ischaemic aetiology and higher plasma levels of N‑terminal pro-B-type natriuretic peptide were all significantly associated with an increase in LVEF in a multivariable linear model. A history of hypertension was associated with a decrease in LVEF.

**Table 4:** Univariable and multivariable predictors of improvement in left ventricular ejection fraction (*LVEF*)

	Model 1 (multivariable)		Model 2 (stepwise)	
	Standardised beta (SE)	*p*-value	Standardised beta (SE)	*p-*value
Men	−0.588 (1.234)	0.63		
Age	−0.043 (0.046)	0.34		
Ischaemic heart disease	−3.105 (1.235)	0.01	−3.790 (1.102)	< 0.01
Hypertension	−1.735 (1.254)	0.17	−1.692 (1.109)	0.13
Dilated cardiomyopathy	0.857 (1.653)	0.60		
Diabetes mellitus	1.904 (1.396)	0.17		
COPD	0.303 (1.697)	0.86		
Atrial fibrillation (history of)	2.692 (1.224)	0.03	2.149 (1.116)	< 0.1
Systolic blood pressure	0.035 (0.030)	0.24		
Weight	−0.030 (0.037)	0.41		
Sodium	−0.199 (0.172)	0.25		
Potassium	0.329 (1.247)	0.79		
Creatinine (log)	−0.192 (1.914)	0.92		
NT-proBNP (log)	−1.071 (0.541)	0.06	−1.021 (0.426)	< 0.05
Heart rate	0.006 (0.035)	0.87		
BB % target dose	0.041 (1.991)	0.98		
ACEI/ARB/ARNI % target dose	−1.588 (1.735)	0.36		
MRA % target dose	−1.026 (1.107)	0.35		
LVEF (baseline)	−0.584 (0.048)	< 0.01	−0.555 (0.043)	< 0.01

*SE* standard error, *COPD* chronic obstructive pulmonary disease, *NT-proBNP* N-terminal pro-B-type natriuretic peptide, *BB* beta-blocker, *ACEI/ARB/ARNI* angiotensin-converting-enzyme inhibitor or angiotensin receptor blocker or angiotensin-receptor-neprilysin inhibitor, *MRA* mineralocorticoid antagonist

### Clinical outcomes

For patients with HFrEF, 1‑year mortality was 10%, and mortality after 3.3 years was 22%. In patients with HFrEF, each 5% increase in LVEF was associated with a hazard ratio of 0.83 (0.72–0.96, *p* = 0.011) for mortality in a Cox proportional hazards model adjusted for age and sex, and a hazard ratio of 0.82 (0.73–0.93, *p* = 0.001) for the combined endpoint of mortality and/or HF hospitalisation (Tab. 5).

**Table 5:** Univariable and multivariable Cox hazard ratios for mortality

*All-cause mortality*	*Unadjusted*	*Adjusted model 1*^*a*^	*Adjusted model 2*^*b*^
Change in LVEF (per 5 units)	0.84 (0.74–0.96) *p* < 0.01	0.83 (0.72–0.96) *p* = 0.011	0.94 (0.91–0.98) *p* = 0.001
*All-cause mortality* *+* *HF hospitalisation*	*Unadjusted*	*Adjusted model 1*^*a*^	*Adjusted model 2*^*b*^
Change in LVEF (per 5 units)	0.83 (0.74–0.93) *p* = 0.001	0.82 (0.73–0.93) *p* = 0.001	0.96 (0.93–0.98) *p* = 0.001

^a^Adjusted for age and sex

^b^Adjusted for age, sex, history of ischaemic heart disease, history of hypertension, history of dilated cardiomyopathy, history of diabetes mellitus, history of chronic obstructive pulmonary disease, history of atrial fibrillation, presence of device, systolic blood pressure, weight, sodium, potassium, creatinine (log), N‑terminal pro-B-type natriuretic peptide (log), heart rate, achieved target dose of beta-blockers, achieved target dose of angiotensin-converting-enzyme inhibitor or angiotensin receptor blocker or angiotensin-receptor-neprilysin inhibitor, achieved target dose of mineralocorticoid antagonist

*LVEF* left ventricular ejection fraction, *HF* heart failure

## Discussion

Employing a protocolised scheme of up-titration in a nurse-led HF clinic in patients with new-onset HF leads to appropriate use of GDMT, which is similar compared to that reported in recent studies [5, 6]. Moreover, reasons for not achieving target doses were recorded in all patients, which was often lacking from previous registries. This approach was accompanied by substantial increases in LVEF in more than one third of patients. Finally, all-cause mortality was in line with the number reported in literature [13].

### Nurse-led HF clinics

Several studies have investigated the effects of nurse-led disease management programmes in HF. Nurse-led care was associated with better patient-reported outcomes in a randomised study from Germany [8]. Planned referral to a nurse-led HF clinic was associated with lower risk of death (but not HF hospitalisation) in the Swedish Heart Failure Registry [9]. Similar results were found in the Dutch Deventer-Alkmaar study [14]. In contrast, in a multi-centre randomised trial (the Which Heart failure Intervention is most Cost-effective in reducing Hospital stay (WHICH? II) Trial) a structured, nurse-led, multidisciplinary management programme did not lead to a reduction in hospitalisation rate or all-cause mortality in Australia. There was, however, a better cardiac recovery on echocardiography (defined as improvement in either LVEF, left ventricular hypertrophy or E/e’) after 3 years of follow-up [15]. In the Coordinating Study Evaluating Outcomes of Advising and Counseling in Heart Failure (COACH) study with 1023 participants, investigating the effect of education and counselling in HF, there was no difference in all-cause mortality or HF hospitalisation after 18 months of follow-up [16]. In this latter study however, up-titration of HF medication was not part of the study protocol. The additive value of a nurse-led HF clinic might therefore depend on the healthcare system where it is initiated. Reasons why nurse-led HF clinics might be superior to standard care are the ability to closely monitor symptoms, optimise treatment by frequent dose adjustments, and the possibility of providing elaborate education on self-care and psychosocial support to patients with HF, including easy access to a healthcare provider in the case of deterioration.

### Factors limiting up-titration

In the present study, we carefully documented reasons for not reaching the recommended target doses in all patients. For beta-blockers, the most common reasons preventing further up-titration were hypotension, or associated complaints such as dizziness or light-headedness, and bradycardia. Hypotension is common in HF, and it can be difficult to distinguish disease-related hypotension from the effect of drugs. A recent meta-analysis found that individual beta-blockers did not exhibit a graded dose-response effect on systolic or diastolic blood pressure over the recommended dose range, suggesting that patients on lower doses might be able to tolerate higher doses as well [17]. In addition, there is evidence from a post hoc analysis of the COPERNICUS trial that patients with the lowest initial blood pressure had the highest improvement in quality of life if treated with carvedilol. In this group of HF patients, blood pressure increased instead of decreasing after treatment with a beta-blocker [18]. For ACEIs/ARBs/ARNIs, other frequent reasons hampering up-titration were renal dysfunction and hyperkalaemia. However, patients with incident hyperkalaemia who are maintained on ACEIs/ARBs/ARNIs might have better survival than those in whom ACEIs/ARBs/ARNIs were down-titrated or stopped because of hyperkalaemia [19].

The ESC HF guidelines provide a class IA recommendation to start with a beta-blocker and RAS inhibitor for all patients with HFrEF. An MRA is indicated in those patients who remain symptomatic despite treatment (NYHA class II or higher) and have a LVEF < 35%. In our cohort of patients with HFrEF, after 9 months of protocolised up-titration, a frequent reason for not receiving the target dose of MRAs was that patients had become asymptomatic (NYHA class I) or that their LVEF had improved. This raises the question whether introducing an MRA earlier, before target doses of beta-blockers and RAS inhibitors are achieved, might lead to improved outcomes. At present, there are few data available to answer this question.

### Improvement in ejection fraction

We found a high percentage of improvement of LVEF in patients with new-onset HFrEF. This percentage is similar to that in a recent retrospective cohort study that found that in 38% of 3124 patients with HFrEF had an increase in LVEF of ≥ 10% after ≥ 6 months of follow-up [3]. In our cohort, patients with ischaemic heart disease were less likely to improve, whereas improvement was more likely in those with a dilated cardiomyopathy. Of note is that LVEF is subject to measurement variation. A study from 2012 concluded that around 20% of patients would be reclassified to a different category if two observers assessed the same echocardiogram and a single cut-off was used [20].

### Strengths and limitations

This study has some limitations. First, this is a single-centre study, which limits the generalisability of the results. The population we studied is on average slightly younger than that of other (larger) HF registries. In addition, by including only patients who were referred to the nurse-led outpatient clinic, HF patients that were being treated by a cardiologist only are not included in the study, which might introduce selection bias. However, other baseline characteristics are very similar to those seen in the literature. Second, because we assessed up-titration and LVEF at 9 months, survivorship bias is introduced since patients that died (*n* = 35) before the follow-up visit were not included.

## Conclusion

Protocolised up-titration of GDMT by a specialised HF nurse as part of a HF management programme leads to doses that are higher than those thus far reported in the literature. This approach was accompanied by an improvement in LVEF in more than one third of patients with new-onset HFrEF. Improvement of LVEF was independently associated with a lower risk of all-cause mortality and HF hospitalisation. This study supports the recommendation to use a specialised HF clinic setting in patients who have been recently diagnosed with HF.

## Supplementary Information

Supplementary table 1: Daily target doses of Guideline-Directed Medical Therapy

## References

[1] Aimo A, Gaggin HK, Barison A, Emdin M, Januzzi JL (2019). Imaging, biomarker, and clinical predictors of cardiac remodeling in heart failure with reduced ejection fraction. JACC Heart Fail.

[2] Gulati G, Udelson JE (2018). Heart failure with improved ejection fraction: is it possible to escape one’s past?. JACC Heart Fail.

[3] Lupón J, Gavidia-Bovadilla G, Ferrer E, de Antonio M, Perera-Lluna A, López-Ayerbe J, Domingo M, Núñez J, Zamora E, Moliner P, Díaz-Ruata P, Santesmases J, Bayés-Genís A (2018). Dynamic trajectories of left ventricular ejection fraction in heart failure. J Am Coll Cardiol.

[4] Savarese G, Vedin O, D’Amario D, Uijl A, Dahlström U, Rosano G, Lam CSP, Lund LH (2019). Prevalence and prognostic implications of longitudinal ejection fraction change in heart failure. JACC Heart Fail.

[5] Greene SJ, Fonarow GC, DeVore AD (2019). Titration of medical therapy for heart failure with reduced ejection fraction. J Am Coll Cardiol.

[6] Brunner-La Rocca H-P, Linssen GC, Smeele FJ (2019). Contemporary drug treatment of chronic heart failure with reduced ejection fraction. JACC Heart Fail.

[7] Marti CN, Fonarow GC, Anker SD (2019). Medication dosing for heart failure with reduced ejection fraction—Opportunities and challenges. Eur J Heart Fail.

[8] Güder G, Störk S, Gelbrich G (2015). Nurse-coordinated collaborative disease management improves the quality of guideline-recommended heart failure therapy, patient-reported outcomes, and left ventricular remodelling. Eur J Heart Fail.

[9] Savarese G, Lund LH, Dahlström U, Strömberg A (2019). Nurse-led heart failure clinics are associated with reduced mortality but not heart failure hospitalization. J Am Heart Assoc.

[10] Ponikowski P, Voors AA, Anker SD (2016). 2016 ESC Guidelines for the diagnosis and treatment of acute and chronic heart failure: The Task Force for the diagnosis and treatment of acute and chronic heart failure of the European Society of Cardiology (ESC). Developed with the special contribution. Eur J Heart Fail.

[11] McMurray JJV, Adamopoulos S, Anker SD (2012). ESC Committee for Practice Guidelines. ESC Guidelines for the diagnosis and treatment of acute and chronic heart failure 2012: The Task Force for the Diagnosis and Treatment of Acute and Chronic Heart Failure 2012 of the European Society of Cardiology. Developed in collaboration with the Heart. Eur Heart J.

[12] Ouwerkerk W, Voors AA, Anker SD (2017). Determinants and clinical outcome of uptitration of ACE-inhibitors and beta-blockers in patients with heart failure: a prospective European study. Eur Heart J.

[13] Shah KS, Xu H, Matsouaka RA, Bhatt DL (2017). Heart failure with preserved, borderline, and reduced ejection fraction: 5-year outcomes. J Am Coll Cardiol.

[14] Bruggink-André De La Porte PWF, Lok DJA, Van Veldhuisen DJ (2007). Added value of a physician-and-nurse-directed heart failure clinic: results from the Deventer-Alkmaar heart failure study. Heart.

[15] Scuffham PA, Ball J, Horowitz JD (2017). Standard vs. intensified management of heart failure to reduce healthcare costs: results of a multicentre, randomized controlled trial. Eur Heart J.

[16] Jaarsma T, Van Der Wal MHL, Lesman-Leegte I (2008). Effect of moderate or intensive disease management program on outcome in patients with heart failure: Coordinating Study Evaluating Outcomes of Advising and Counseling in Heart Failure (COACH). Arch Intern Med.

[17] Wong GW, Boyda HN, Wright JM (2016). Blood pressure lowering efficacy of beta-1 selective beta blockers for primary hypertension. Cochrane Database Syst Rev.

[18] Rouleau JL, Roecker EB, Tendera M (2004). Influence of pretreatment systolic blood pressure on the effect of carvedilol in patients with severe chronic heart failure: the Carvedilol Prospective Randomized Cumulative Survival (COPERNICUS) study. J Am Coll Cardiol.

[19] Beusekamp JC, Tromp J, Cleland JGF (2019). Hyperkalemia and treatment with RAAS-inhibitors during acute heart failure hospitalizations and their association with mortality. JACC Heart Fail.

[20] Kaufmann BA, Min SY, Goetschalckx K (2013). How reliable are left ventricular ejection fraction cut offs assessed by echocardiography for clinical decision making in patients with heart failure?. Int J Cardiovasc Imaging.

