# Supplementary material for: Improvement in left ventricular ejection fraction after pharmacological up-titration in new-onset heart failure with reduced ejection fraction
Source: Neth Heart J. 2021 Jun 14;29(7-8):383–93. doi: 10.1007/s12471-021-01591-6 (PMC8271074; doi:10.1007/s12471-021-01591-6)
Supplement: Supplementary file 1 — Supplementary table 1: Daily target doses of Guideline-Directed Medical Therapy [file 12471_2021_1591_MOESM1_ESM.docx]

Supplementary table 1: Daily target doses of Guideline-Directed Medical Therapy

| **Angiotensin-converting enzyme inhibitors** |  |
| --- | --- |
| captopril | 150 mg |
| enalapril | 20 mg |
| lisinopril | 20 mg |
| ramipril | 10 mg |
| perindopril | 8 mg |
|  |  |
| **Angiotensin receptor blockers** |  |
| candesartan | 32 mg |
| valsartan | 320 mg |
| losartan | 150 mg |
|  |  |
| **Angiotensin receptor-neprilysin inhibitor** |  |
| sacubitril/valsartan | 194 / 206 mg |
|  |  |
| **Beta-blockers** |  |
| bisoprolol | 10 mg |
| carvedilol | 50 mg |
| metoprolol succinate | 200 mg |
| nebivolol | 10 mg |
|  |  |
| **Mineralocorticoid antagonist** |  |
| eplerenone | 50 mg |
| spironolactone | 25 mg |
